# Study on the Preparation and Performance of Lightweight Wallboards from MSWIBA Foam Concrete

**DOI:** 10.3390/ma17174402

**Published:** 2024-09-06

**Authors:** Yun Dong, Yao Wang, Zhancheng Zhou, Haoyue Fan

**Affiliations:** 1 Faculty of Architecture and Civil Engineering, Huaiyin Institute of Technology, Huai’an 223001, China; dyun@hyit.edu.cn; 2 College of Water Conservancy and Hydropower Engineering, Hohai University, Nanjing 210098, China; 3 Department of Civil Engineering, Nanjing Institute of Technology, Nanjing 210098, China

**Keywords:** MSWIBA, foam concrete, lightweight wall panels, sound insulation performance

## Abstract

To reduce land use and avoid further pollution, incineration for power generation has become the main method for municipal solid waste treatment. This research focused on the potential for transforming Municipal Solid Waste Incineration Bottom Ash (MSWIBA) into a finely ground powder. The impact of the powder’s fineness and the amount of water used on its effectiveness was analyzed using a method called grey theory. MSWIBA was used as a partial substitute for cement in making MSWIBA foam concrete and lightweight wall panels. By modifying the fineness and water utilization of the recycled micro-powder, its maximum activity index can be increased to 90.1. This study determined the influence of factors including apparent dry density, water–cement ratio, foaming agent dilution ratio, and admixture dosage on the strength of the recycled foam concrete, and established the optimal mix ratio. This study employed a combination of physical experiments and numerical simulations to elucidate the impact of panel material, core layer thickness, and layer sequence on sound insulation performance. The simulation results were in close agreement with the experimental findings.

## 1. Introduction

Rapid urbanization and economic development have led to increased consumption of resources and production of waste. Incineration is a primary waste treatment method due to its efficiency in volume reduction and energy recovery. Municipal solid waste incineration bottom ash (MSWIBA) [1,2] contains a mix of inorganic compounds, metals, and residual organics. Presently, MSWIBA is utilized mainly in two ways [3]: (a) as landfill cover material [4], reducing environmental and health risks; (b) as substituting materials like gravel in road or concrete construction [5]. However, in some countries, MSWIBA is classified as hazardous due to toxic elements such as dioxins and heavy metals. Stabilized landfill, often used to treat IFA, consumes substantial land and may cause secondary pollution to water and soil. Previous studies indicate [6] that SiO_2_, Al_2_O_3_, and CaO are the primary chemical components in MSWIBA, making up about 75% of its total weight. This typical CaO-SiO_2_-Al_2_O_3_ chemical system shares similar hydration properties with cement and fly ash [7,8]. Despite its potential, there is limited research on the hydration activities of MSWIBA and its value when recycled, especially as an alternative material in concrete [9]. This project focuses on ultra-finely grinding MSWIBA to enhance its strength and increase its value, aiming to provide substantial economic and social benefits.

Foam concrete [10] is a lightweight [11], porous [12] material made by introducing uniformly distributed bubbles into cement paste, featuring good thermal and acoustic insulation properties and low density. Previous studies indicate [13,14] that the performance of foam concrete is influenced by variables such as the proportion of fly ash, volume of foam, and characteristics of the pores. Kearsley et al. [13,15] found that a high proportion of fly ash in place of cement reduces early strength but has a minimal impact on long-term strength. Furthermore, an appropriate quantity of fly ash can improve the workability of the cement paste [16,17]. Nambiar et al. [18,19] identified that the type of aggregate and amount of foam are key factors; reducing the size of coarse aggregate particles augments mechanical properties, with pore spacing playing a prominent role in affecting the mechanical properties and dry density. Jones et al. [20] observed that replacing some of the natural sand with low-calcium fly ash notably enhances the workability of the slurry and long-term mechanical properties. Preliminary research indicates that MSWIBA exhibits pozzolanic activity, and incorporating small portions of pozzolanic materials does not radically change the overall performance of foam concrete. Therefore, this research is directed towards examining the fineness of recycled micro-powder grinding and assessing the performance of foam concrete using this recycled micro-powder.

Foam concrete is characterized by its uniform pore structure, which is formed through the use of gas-producing chemicals or foaming agents. Foam serves as a porosity-forming agent [21,22], resulting in a porous solid when mixed with other raw materials. Compared to traditional Portland cement concrete, the bubbles reduce density [23]. This results in specific properties [24] like excellent sound [25] and thermal insulation [26]. Currently, developing high-quality soundproof materials [27,28] is a hotspot in noise control research, particularly for use in lightweight wall panels that consist of multiple layers of soundproof or sound-absorbing materials. Toshio et al. [29] found that increasing the number of layers from two to four improves the soundproofing performance of panels. Carneal et al. [30] suggest that altering the composite structure of the material can enhance its acoustic performance. Their research notes that soundproofing at low frequencies tends to be less effective, following the “mass law”, but this can be remedied by designing varied structures. Yilmazer et al. [31] believe that that incorporating expanded perlite particles into wall panels boosts both their strength and sound absorption qualities. Valle et al. [32] developed a new porous soundproof board using aluminum slag, marble slag, foundry sand, recycled polystyrene, and plastic clays, achieving over 95% soundproofing at 500 Hz. Thus, the industrial application of foam concrete in soundproof wall panels is another focus of this study.

In this research, ground micro-powder made from MSWIBA was used as a substitute for cement to create recycled foam concrete and explores its basic properties. The primary goal was to use this material as the core layer in wall panels to enhance the use of MSWIBA resources. The principal contents of the research are as follows:

(1) Preparation and activation of recycled micro-powder: MSWIBA was ground into a fine powder to replace cement. The optimal particle size, water amount, and replacement range were determined through the utilization of grey relational analysis and tests.
(2) Recycled foam concrete development: Single-factor tests examined the impact of various factors, including apparent dry density, water–cement ratio, foaming agent dilution, and admixture content, on compressive strength and water absorption. The optimal mix ratio was determined by orthogonal tests.
(3) Sound insulation performance of recycled lightweight wall panels: Using recycled foam concrete as the insulation layer in composite panels, physical tests and COMSOL simulations studied the effects of core layer order, surface material, and wall thickness on sound insulation.

## 2. Materials and Methods

### 2.1. Materials

#### 2.1.1. Municipal Solid Waste Incineration Bottom Ash (MSWIBA)

**Figure 1:**
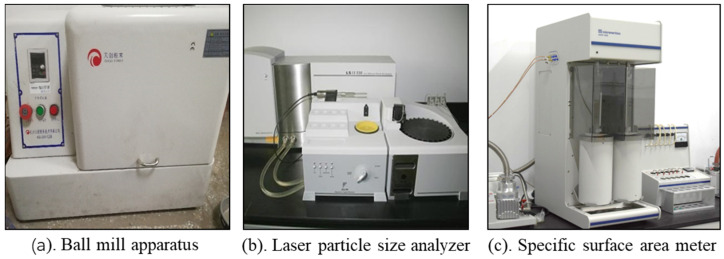
Experimental facilities.

The MSWIBA used in this study was sourced from a municipal solid waste incineration power plant in China and primarily consists of slag, ferrous metals, ceramic particles, and non-combustible materials. Subsequently, an SK-2 ball mill (Changsha, China, Changsha Tianchuang Powder Technology Co., Ltd.) was employed for the grinding of the MSWIBA, followed by particle size analysis with a Bettersize2000 laser analyzer (Dandong, China, Dandong Baite Instrument Co., Ltd.). The specific surface area was determined using a specific surface area analyzer (HeFei, China, HeFei Guoyi Quantum Technology Co., Ltd.). The equipment used in the experiment is shown in Figure 1.

**Figure 2:**
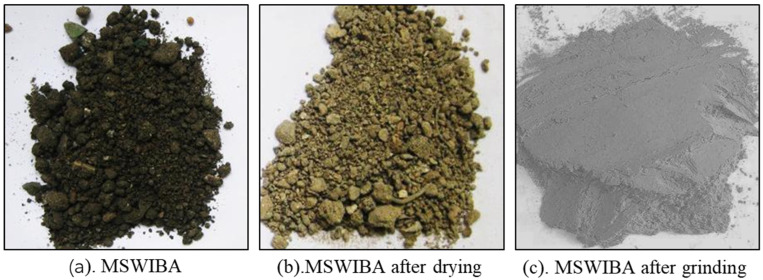
Sample of MSWIBA. (**a**) Original sample. (**b**) Sample after drying. (**c**) Grinded sample.

The selected samples, processed through screening, crushing, and washing, displayed rough grey-black surfaces that turned light brown upon drying and emitted a mild odor (as shown in Figure 2). The MSWIBA was ground using a ball mill to produce eight types with specific surface areas labeled P1 to P8. The specific surface areas of the materials are presented in Table 1. The primary chemical components in MSWIBA are Si, Ca, and Al, with small amounts of Fe and Mg, mostly existing as free oxides, as detailed in Table 2.

#### 2.1.2. Foam Concrete Preparation Materials

The concrete tests used Conch brand P·O42.5 Portland cement (Huaian, China, Anhui Huaian Conch Cement Co., Ltd.), with an ISO standard sand particle size ranging from 0.08 to 2 mm. Detailed performance indicators of the AES polymer composite foaming agent (Langfang, China, Langfang XingRUI TECHNOLOGY Development Co., Ltd.) used in the tests are shown in Table 3. The polycarboxylate superplasticizer (CQJ-JSS02) was provided by Shanghai Chenqi Chemical Technology Co., Ltd. (Shanghai, China). Industrial-grade anhydrous sodium carbonate (Tianjin, China, Tianjin Zhiyuan Chemical Reagent Co., Ltd.) served as the accelerator, while triethanolamine (Yatai, China, Wuxi Yatai UNITED CHEMICAL Co., Ltd.) was used as the reinforcing agent.

### 2.2. Experimental Design and Methods

#### 2.2.1. MSWIBA Performance Test Pre-Experiment

(1) Mortar Strength Test

This experiment was designed based on the preparation method of cement mortar, where MSWIBA substituted 30% of the cement to form mortar, with resultant strength assessments to follow. The mold dimensions were 40 mm × 40 mm × 160 mm, with 24 h of moisture curing and 28 days of water curing. The experiment designs are presented in Table 4, including a control group N_a_-C1 with 70% cement and no micro-powder, and another control group N_a_-C2 with 100% cement and no micro-powder.

(2) Fineness Analysis Based on Grey Theory

The particle size distribution of MSWIBA is closely linked to its strength activity, which subsequently influences the compressive strength of mortar. In order to ascertain the optimal fineness range, this chapter employed the technique of grey relational analysis [33,34] to investigate the correlation between the fineness of recycled bottom ash micro-powder and its mortar strength activity. Grey relational analysis is a technique employed to evaluate the extent of influence exerted by disparate factors (subsequences) on the overall system (main sequence). In a system comprising multiple factors, the factors may exhibit similar or opposite trends. Grey relational analysis enables the quantification of the degree of similarity or dissimilarity between these factors by calculating their degree of association. The degree of association serves to quantify the connections between variables, whereby higher values represent stronger correlations. The calculation method is as follows:

(1) $$\begin{array}{c} \xi_{i}(k)=\frac{\min_{i=1}^{n}\min_{k=1}^{m}\left|x_{o}(k)-x_{i}(k)\right|+\sigma \cdot \max_{i=1}^{n}\max_{k=1}^{m}\left|x_{o}(k)-x_{i}(k)\right|}{\left|x_{o}(k)-x_{i}(k)\right|+\sigma \cdot \max_{i=1}^{n}\max_{k=1}^{m}\left|x_{o}(k)-x_{i}(k)\right|}\\ i=0,1,2,\cdots ,n;k=1,2,3,\cdots ,m \end{array}$$

where $\xi_{i}(k)$ is the correlation coefficient of the *k*–th subsequence, $x_{i}(k)$ is the *k*–th index of the *i*–th factor, $\min_{i=1}^{n}\min_{k=1}^{m}\left|x_{o}(k)-x_{i}(k)\right|$ is the minimum difference in the subsequence, and $\max_{i=1}^{n}\max_{k=1}^{m}\left|x_{o}(k)-x_{i}(k)\right|$ is the maximum difference in the subsequence. σ is the resolution coefficient, which is generally in the range of (0, 1), here set at 0.5. The relationship between mean data sequences and the parent sequence is called the degree of association, denoted as follows:

(2) $$S_{i}=\frac{1}{m}\sum_{k=1}^{m}\xi_{i}(k)$$

In the calculation of the degree of association, the absolute difference is used instead of the direct difference, eliminating the ability to discern the association’s polarity among factors, particularly whether they demonstrate positive or negative correlations. To better distinguish the correlation, the positive–negative pole method is introduced, as explained below:

(3) $$\begin{array}{c} \theta_{i}=\sum_{k=1}^{n}kx_{i}(k)-\frac{\sum_{k=1}^{n}x_{i}(k)\sum_{k=1}^{n}k}{n}\\ i=0,\theta_{i}=\theta_{0} \end{array}$$

(4) $$\theta_{k}=\sum_{k=1}^{n}k^{2}-\frac{(\sum_{k=1}^{n}k{)}^{2}}{n}$$

If $\mathrm{sgn}(\frac{\theta_{i}}{\theta_{k}})=\mathrm{sgn}(\frac{\theta_{0}}{\theta_{k}})$, then $x_{0}(k)$ and $x_{i}(k)$ are positively correlated. If $\mathrm{sgn}(\frac{\theta_{i}}{\theta_{k}})=-\mathrm{sgn}(\frac{\theta_{0}}{\theta_{k}})$, $x_{0}(k)$ and $x_{i}(k)$ are negatively correlated.

(3) Standard Consistency Water Test

The fluidity test assesses the compatibility of cementitious particles, reflecting the water requirement for the MSWIBA–cement mixture. According to China NCSTC Standard GB175-2007 [35], the fluidity of mixed material mortar must reach a minimum of 180 mm. When measuring fluidity, standard sand acts only as an aggregate without hydration reaction. The theoretical water use for MSWIBA mortar is calculated by summing up the water consumption of cement with the water demand of MSWIBA, as demonstrated below:

(5) $$V=\frac{\frac{P_{C}}{P}\times C\times m_{s}+C\times m_{c}}{\rho }$$

#### 2.2.2. Substitution Ratio of MSWIBA

To investigate the impact of various MSWIBA contents on mortar strength, 11 experiments were conducted with different substitution ratios as outlined in Table 5. The experiments maintained a consistent water–cement ratio and featured MSWIBA with a specific surface area of 800–900 m^2^/kg (P6). MSWIBA was used to replace cement in proportions ranging from 5% to 50%. D11 was designated as the control group.

#### 2.2.3. Single-Factor Mix Design of MSWIBA Foam Concrete

By managing variables, the impact of different factors on the performance of recycled foam concrete was examined. In the single-factor ratio design approach, the key data points that required alterations included the following: total dry material mass, water to cement ratio, foaming agent dilution rate, and admixture content. Throughout these experiments, three factors were held steady, whereas one factor was modified, with the MSWIBA content fixed at 10%. Table 6, Table 7, Table 8 and Table 9 present the experimental design schemes for varying variable factors.

#### 2.2.4. Orthogonal Test of MSWIBA Foam Concrete

This research established a three-factor, three-level orthogonal experiment to assess the 28-day compressive strength and water absorption characteristics of MSWIBA foam concrete. The evaluation considered three primary factors: apparent dry density, water–cement ratio, and foaming agent dilution rate. The orthogonal experimental set the values for apparent dry density at [350 kg/m^3^, 450 kg/m^3^, 550 kg/m^3^], the water–cement ratio at [0.4, 0.45, 0.5], and the foaming agent dilution at [1:20, 1:40, 1:60]. Table 10 shows 9 experimental groups with different factor combinations and the same MSWIBA content.

## 3. Results and Discussion

### 3.1. Analysis of MSWIBA Pre-Experimental Results

#### 3.1.1. MSWIBA Mortar Strength

**Figure 3:**
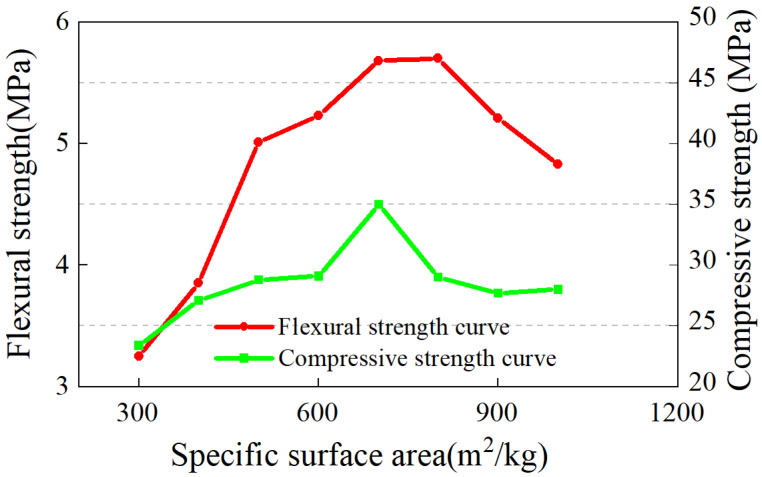
Variation of mortar strength with specific surface area of MSWIBA.

Figure 3 shows the 28-day compressive and flexural strengths of P1~P9 mortar. The strength of the mortar initially rose and then declined with an increase in the MSWIBA specific surface area. At point P5, the compressive strength reached its maximum at 35.0 MPa, and the flexural strength peaked at 5.68 MPa. As the surface area continued to increase, the compressive strength began to decrease, whereas flexural strength increased slightly to 5.7 MPa at P6, then subsequently fell.

#### 3.1.2. Correlation between Fineness and Mortar Properties

**Figure 4:**
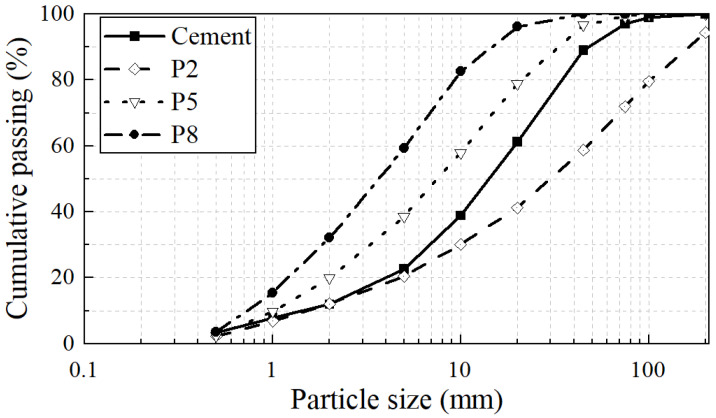
Particle size analysis results.

The particle size distribution of MSWIBA with specific surface areas of P2, P5, and P8, as well as cement particles, was measured using the Bettersize2000 laser particle size analyzer, as shown in Figure 4. The test outcomes were methodically classified: the 3-day and 28-day compressive strengths of the MSWIBA mortar were designated as the parent sequence, while the percentage of various particle groups within MSWIBA was labeled as the subsequence, as shown in the Table 11.

It can be observed that the correlation order of different particle groups of MSWIBA was consistent, with particles in the 5–20 μm range showing the strongest correlation with mortar strength. Particles smaller than 20 μm exhibited a positive correlation with mortar strength, while those larger than 20 μm inhibited the activation of micro-powder and the enhancement of mortar strength. Consequently, the particle size distribution within the micro-powder had a significant impact on the 3-day and 28-day compressive strength of the mortar. Increasing the content of particles in the 5~20 μm range markedly enhanced the activity of the micro-powder. Therefore, by controlling the sieve mesh size to 200 mesh (average particle size between 0.200 mm and 0.075 mm), the activity of MSWIBA-recycled micro-powder could be greatly enhanced, thereby improving the compressive strength of the mortar.

#### 3.1.3. Optimization of Water Consumption in MSWIBA Strength Test 

In accordance with the fluidity test results presented in Table 12, the total water volume calculated using the mortar formula fails to meet the standard for fluidity of greater than 180 mm. To fulfill the fluidity criterion, it is necessary to add at least 199.1 mL of water. Moreover, the overall water quantity must also satisfy the minimum requirement for the standard consistency of the paste.

The fluidity of mortar incorporating MSWIBA as a replacement for cement should be equivalent to that of pure cement mortar. To accomplish this, this study refined the calculation formula for the total water volume by incorporating a correction factor. The calculation method is as follows:

(6) $$V=\frac{\frac{P}{P_{C}}\times C\times m_{s}+C\times m_{c}}{\rho }\times \mu$$

where correction factor μ is 1.11~1.14, and when *V* is greater than 225 mL, it is taken as 225 mL.

### 3.2. Improved MSWIBA Characteristics Comparison and Replacement Ratio Test

#### 3.2.1. Improved MSWIBA Feature Comparison

**Figure 5:**
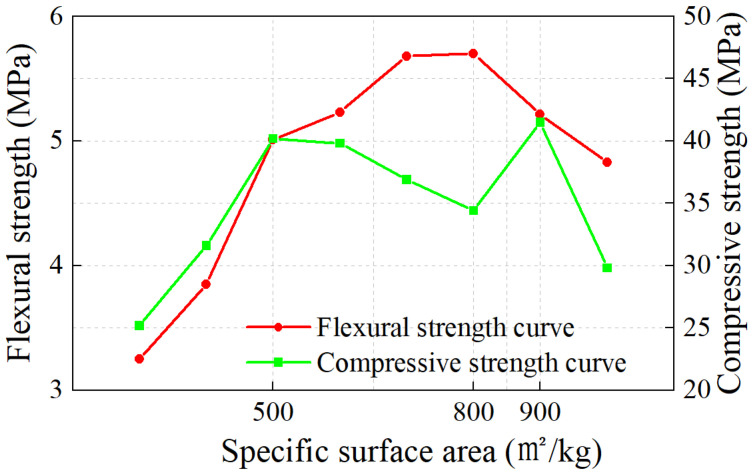
Improved mortar strength test.

According to the water demand formula, the total water amount in Table 4 was adjusted from 225 g to 201.6 g for retesting (Test group-N_b_), as shown in Figure 5. It can be observed that when the specific surface area was 800~900 m^2^/kg, the interaction between coarse and fine particles created a complementary balance within the mortar system, achieving a maximum flexural strength of 5.74 MPa. This indicates that when the specific surface area difference between cement and MSWIBA is mineral, the elastic moduli of the hydration products are similar, which promotes interface bonding. The compressive strength exhibited a four-stage characteristic, achieving the initial peak of 40.2 MPa at a specific surface area of 500~600 m^2^/kg. Upon the specific surface area reaching 900~1000 m^2^/kg, it attained a second peak of 41.5 MPa.

Table 13 shows that the strength activity index of most of the MSWIBA exceeded 50%, with the highest reaching 74.9%. By comparing samples N_a_ and N_b_, it is evident that a reduction in water content corresponded with an increase in strength activity, noted by a 23.5% increase in sample P7. This suggests that when the fineness of MSWIBA particles is excessively coarse, they fail to achieve optimal dense packing. Variations in specific surface area lead to the overconsumption of water, creating a molecular water film around the particles, with hydration reactions predominantly occurring on the MSWIBA particle surfaces. For the N_b_ test group, when the specific surface area of MSWIBA and cement is similar, the particle size tends to be consistent, the hydration reaction is sufficient, and the compressive strength of the mortar is improved. A specific surface area of 900–1000 m^2^/kg represents the optimal particle size distribution, which can enhance the density of mortar and achieve maximum compressive strength. However, as the specific surface area is increased further, the rise in fine particles results in internal voids and stress concentrations, which in turn lead to a decline in compressive strength. This suggests that when the difference in specific surface area is minimal, the elastic modulus of the hydration products is comparable, leading to enhanced interfacial bonding and improved flexural strength. Conversely, when the difference is too large, the process of interface bonding becomes challenging, porosity rises, and flexural strength declines. Consequently, through adjustments in water usage and particle fineness, the potential activity of MSWIBA can be activated, thus enhancing the quality of the mortar.

**Figure 6:**
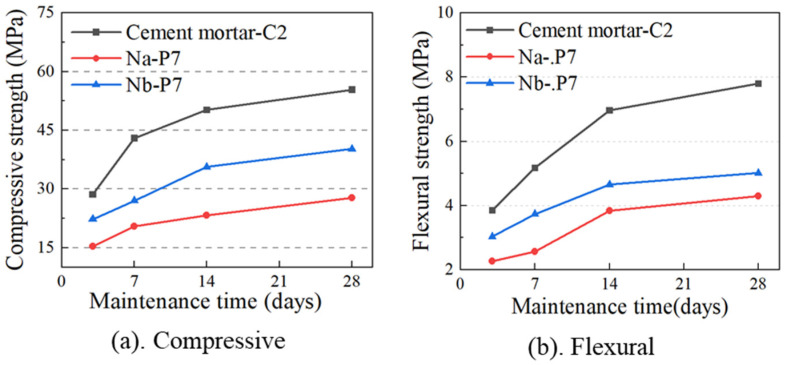
The trend of MSWIBA mortar strength with age, (**a**) Compressive strength growth curve, (**b**) Flexible strength growth curve.

The strength growth curve of MSWIBA mortar with a specific surface area of 800~900 m^2^/kg before and after adjusting the water amount is shown in Figure 6. The results indicate that modifying the water amount leads to an increment in the compressive strength of the mortar across various curing periods, exhibiting a trend similar to that of cement. As the curing period progresses, the growth rate of compressive strength gradually decreased and reached a steady value, similar to the trend observed in flexural strength. This suggests that during the hydration process, the active component content in MSWIBA is lower compared to that in cement, and excess water hampers gel formation, leaving active components unbound and prone to forming microcracks. Thus, altering the water amount substantially influences the mortar’s strength, although it does not affect the hydration reaction or the setting time.

#### 3.2.2. MSWIBA Substitution Ratio Test

**Figure 7:**
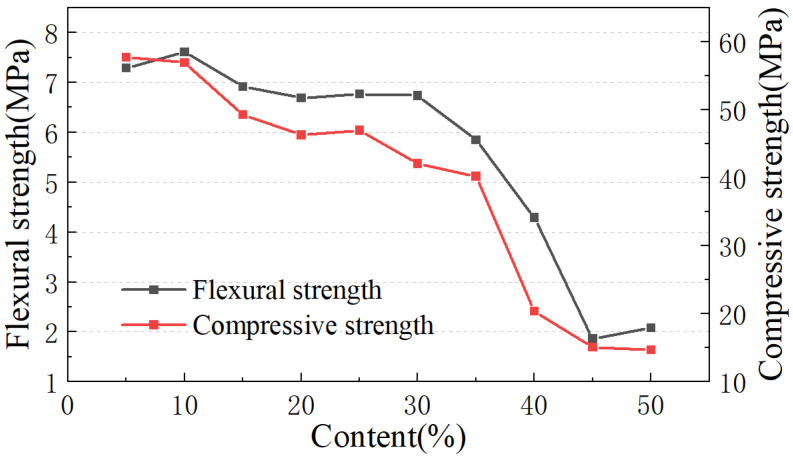
Strength test of mortar with different dosage of MSWIBA.

Figure 7 presents the 28-day compressive and flexural strength for various substitution ratios of MSWIBA. As the substitution amount of MSWIBA increased, the compressive strength of the mortar decreased at each curing period. For flexural strength, aside from a slight increase when the MSWIBA content was between 10% and 30%, the overall trend also decreased. Once the content exceeded 35%, both compressive and flexural strength of the mortar suffered considerable reductions. This degradation can be attributed to the low activity of MSWIBA. After the hydration reaction, the particles tend to cluster, detrimentally impacting the overall strength of the mortar.

According to Table 14, when the substitution amount ranged from 5% to 35%, the corresponding activity index remained slightly above 60%. This is attributed to the fact that as the proportion of active particles around 75 μm increases, voids develop during the hardening process of the mortar, consequently impacting its 28-day strength. When the substitution amount exceeds 35%, the internal voids coalesce to form cracks, precipitating a sharp decline in the mortar’s strength. 

The experiment demonstrated that as the MSWIBA replacement ratio increased, the volume shrinkage of the mortar specimens also increased, resulting in a slower hydration reaction and a reduction in mortar strength. When the replacement ratio was below 10%, the impact on the workability and strength of the mortar was minimal. At a 10% replacement ratio with a specific surface area of 800–900 m^2^/kg, the flexural strength of the mortar reached its peak at 7.6 MPa, while the compressive strength is slightly lower than that of the 5% replacement. As the substitution amount of MSWIBA increases, mortar specimens exhibit increased volume shrinkage, which in turn slows down the hydration reaction and reduces the mortar’s strength. In summary, the optimal strategy for utilizing MSWIBA involves employing a 200-mesh sieve (0.075 mm) to select micro-powder with a specific surface area of 800~900 m^2^/kg and maintaining the substitution amount in concrete products at approximately 10%. This approach ensures the mechanical properties of the mortar while maximizing the resource utilization of MSWIBA.

### 3.3. Single-Factor Test Results and Analysis of MSWIBA Foam Concrete

#### 3.3.1. Effect of Apparent Dry Density on MSWIBA Foamed Concrete

**Figure 8:**
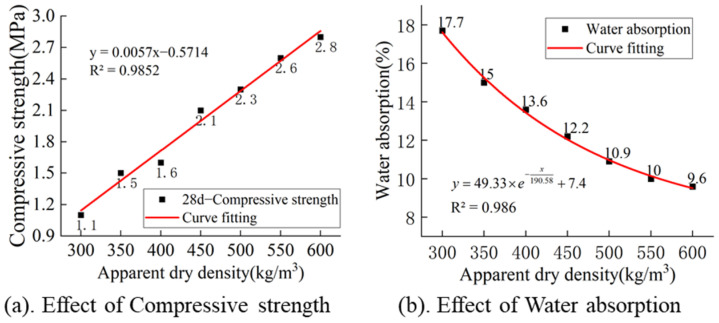
Effect of apparent dry density, (**a**) Effect of apparent dry density on compressive strength, (**b**) Effect of apparent dry density on water absorption.

According to Figure 8a, there exists a strong positive linear correlation between the apparent dry density and the 28-day compressive strength of MSWIBA foamed concrete, with R^2^ = 0.9852. This indicates that an increase in apparent dry density leads to a denser internal structure of the test block, an enhanced solid-phase conversion rate of the cement, and smaller pores, which collectively contribute to an increase in compressive strength. Referencing Figure 8b, it can be observed that as the apparent dry density increases, the water absorption rate of the foamed concrete gradually decreases. This decrease can be attributed to the reduction in size and connectivity of internal voids within the test block, resulting in a lower water absorption rate. 

#### 3.3.2. Effect of Water–Cement Ratio

**Figure 9:**
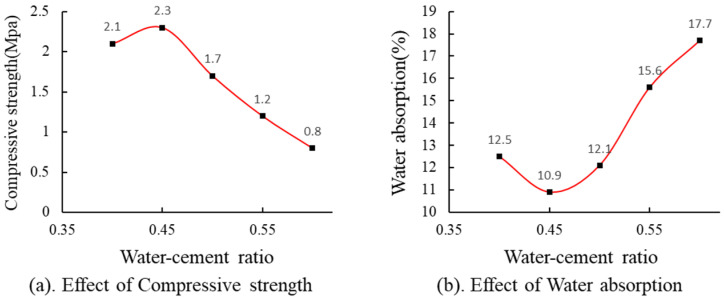
Effect of water–cement ratio, (**a**) Effect of water-cement ratio on compressive strength, (**b**) Effect of water-cement ratio on water absorption.

According to Figure 9a, the strength of MSWIBA foamed concrete peaked at 2.3 MPa when the water–cement ratio was 0.45, demonstrating an overall upward and then downward trend. As indicated in Figure 9b, the water absorption rate attained a maximum of 17.7% at a water–cement ratio of 0.6, exhibiting a pattern of initial decrease followed by an increase. This observation suggests that during the transition from the liquid to solid phase in cement, only a portion of the water engages in the hydration reaction. The surplus free water contributes to the formation of continuous capillary pores or through-pores during the hardening process. These pores facilitate pathways for water ingress into the test block, leading to higher water absorption and reduced density.

#### 3.3.3. Effect of Dilution Ratio

**Figure 10:**
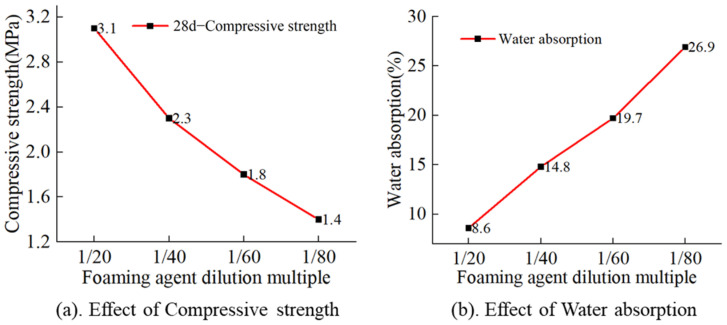
Effect of dilution ratio, (**a**) Effect of dilution ratio on compressive strength, (**b**) Effect of dilution ratio on water absorption.

From Figure 10a, it can be seen that the 28-day compressive strength of MSWIBA foamed concrete showed a decreasing trend as the foaming agent dilution ratio increased. The compressive strength reached 3.1 MPa at a dilution ratio of 1:20; however, it declined to 1.2 MPa at a dilution ratio of 1:80, falling below the standard value. According to Figure 10b, the water absorption rate of MSWIBA foamed concrete augmented with an increase in the foaming agent dilution ratio. This phenomenon occurs because as the water content rises, the closed bubbles formed by the foam inside the concrete expand, disrupting the water molecule equilibrium. This disruption causes internal bubbles to shrink and merge, forming larger pores, which in turn results in a reduction in strength and an increase in water absorption rate.

#### 3.3.4. Effect of Admixtures

From Table 15, it can be seen that additives affected the compressive strength of MSWIBA foamed concrete. Specifically, the combination of 2.5% Na_2_CO_3_, 0.5% water-reducing agent, and 0.05% triethanolamine significantly enhanced the compressive strength of MSWIBA foamed concrete.

### 3.4. Orthogonal Test Results and Analysis

Orthogonal tests were conducted to study the effects of single factors on the 28-day compressive strength and water absorption of MSWIBA foamed concrete. These tests resulted in nine sets of interrelated test results, as displayed in Table 16.

The range analysis method was utilized to further validate the accuracy of the single-factor test results and to ascertain the optimal combination of various factors. By keeping the α factor (apparent dry density) constant, its influence on the test results became more pronounced. When the α factor was set at 350 kg/m^3^, its impact was observed in tests 1, 2, and 3, with K_1_ being the sum of the three indices for α_1_, denoted as K_α1_. As shown in Table 17, the sum of the indices for the three factors, apparent dry density (α), water–cement ratio (β), and foaming agent dilution ratio (γ), at three levels can be calculated: K_1_, K_2_, K_3_. The range value R is the difference between the maximum and minimum values of K_i_ (i = 1, 2, 3), and k_i_ represents the average of K_i_.

**Figure 11:**
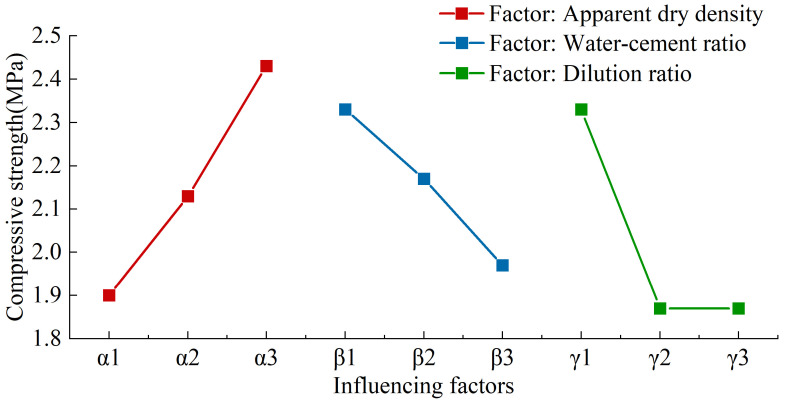
Relationship between influencing factors and 28d-compressive strength.

**Figure 12:**
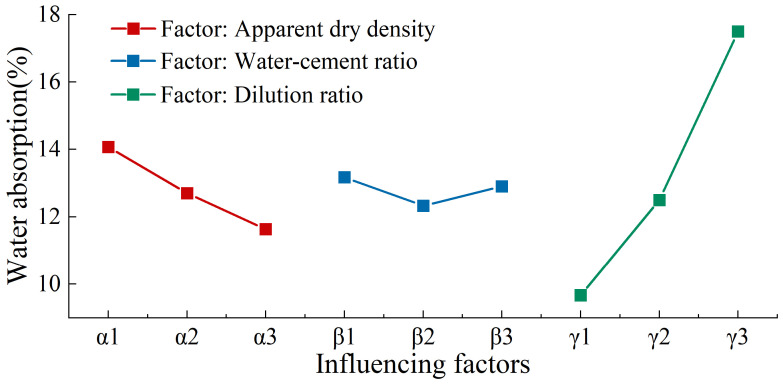
Relationship between influencing factors and water absorption.

From Table 17 and Figure 11 and Figure 12, it is evident that exploring the effects of apparent dry density, water–cement ratio, and foaming agent dilution ratio on the performance of MSWIBA foamed concrete requires a comprehensive consideration of the balance between these factors. The apparent dry density predominantly influenced the 28-day compressive strength and water absorption rate. Therefore, in the design of MSWIBA foamed concrete, appropriately increasing the apparent dry density can significantly improve the compressive strength and reduce the water absorption rate. As the water–cement ratio rose from 0.4 to 0.5, the 28-day compressive strength gradually declined, whereas the water absorption rate initially increased before subsequently decreasing. In the preparation of foamed concrete, the water-to-cement ratio is a critical parameter. A low water-to-cement ratio will result in an insufficient consistency, which will cause the formation of foam prior to the hydration reaction. This may result in the collapse of the mold. Conversely, if the ratio is excessive, the presence of excess free water will impair the stability of the foam, and the evaporation of moisture may result in the formation of internal cracks in the specimens, thereby reducing their strength. Therefore, the water-to-cement ratio should be optimized to ensure proper specimen molding while maintaining mechanical performance. As the dilution ratio of the foaming agent increased, the 28-day compressive strength of MSWIBA foamed concrete decreased and then stabilized, while the water absorption rate gradually increased. This is because the dilution of foaming agent causes the foam cell walls to become thinner, which affects strength and increases the water absorption rate. However, the subsequent stabilization of the 28-day compressive strength may be due to the combined influence of various factors.

Therefore, in mix design, the apparent dry density should be the primary consideration, followed by the foaming agent dilution ratio and the water–cement ratio. From the above analysis, the order of factors affecting the 28-day compressive strength was α > γ > β. The priority of factors affecting the water absorption rate was γ > α > β. High water absorption increases the density and weight of foamed concrete while also diminishing its thermal insulation, soundproofing, and mechanical properties. It is evident that in order to achieve a high compressive strength and low water absorption in foamed concrete, the mix design should prioritize apparent dry density, with secondary considerations being given to the foaming agent’s dilution ratio and the water-to-cement ratio. Through detailed balance analysis and prioritizing strength, the optimal formula was determined to be α_3_β_2_γ_1_, which equates to an apparent dry density of 550 kg/m^3^, a water–cement ratio of 0.4, and a foaming agent dilution ratio of 1:20. In this research, only comparative tests were conducted for additives, showing an improvement in 28-day compressive strength compared to samples without additives. Therefore, additives need to be considered in the mix design, as shown in Table 18. With the optimal mix ratio established, preparation tests of MSWIBA foamed concrete were performed, achieving a 28-day compressive strength of 3.4 MPa and a water absorption rate of 9.6%, both satisfying the specification requirements.

## 4. Study on the Sound Insulation Performance of MSWIBA Foam Concrete Lightweight Wall Panels

### 4.1. Sound Insulation Performance Comparison

#### 4.1.1. Experimental Program of Sound Insulation Performance

**Figure 13:**
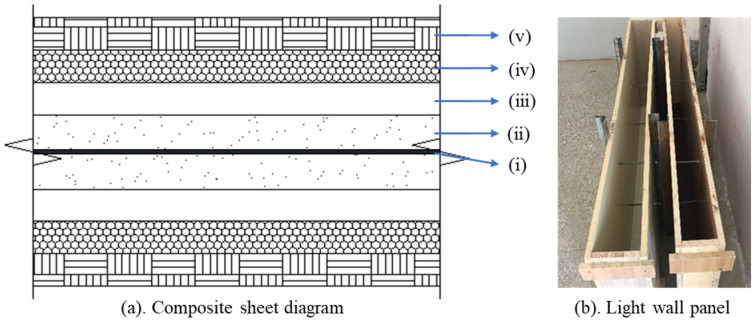
Wall panel model, (**a**) composite sheet diagram: (i) middle perforated panel, (ii) micro-powder foam concrete, (iii) polystyrene foam board, (iv) damping layer, (v) panel, (**b**) light wall panel.

To further enhance the added value of MSWIBA, foamed concrete has been utilized as the core layer of recycled lightweight wall panels, which offer superior sound insulation, lightness, and convenience. The core was manufactured using the optimal mix ratio of MSWIBA foamed concrete. As shown in Figure 13a, the multi-layer composite panel includes a core layer of MSWIBA foamed concrete, a core layer of foam board, a perforated plate in the middle of the core layer, damping layers made of fire-resistant materials on both sides of the core layer, and an outer layer. According to the standard ‘Foamed Concrete Wall Panels’ (JC/T 2475-2018) [36], the wall panel dimensions selected for this test were 1400 mm in length, 600 mm in width, and 90 mm in thickness, as shown in Figure 13b.

The wall panels enhance sound insulation performance through a multi-layer structure composed of different materials. As detailed in Table 19, this research explores the variations in sound insulation performance across different wall panels by modifying the layer arrangement, the thickness of the foamed concrete, and the type of material used for the outer layer. Given that lightweight wall panels behave as volumetric response materials, the local sound insulation is consistent with the overall sound insulation. In this study, the lightweight wall panels were cut into cylindrical specimens with a diameter of 95 mm and a thickness of 90 mm. The AWA6122A standing wave tube served as the testing instrument, while the impedance tube method (the standing wave ratio method) was utilized to assess the sound absorption coefficient and acoustic impedance of the sound-absorbing materials.

#### 4.1.2. Sound Insulation Performance Test Results and Analysis

(1) Effect of core thickness on sound insulation performance

**Figure 14:**
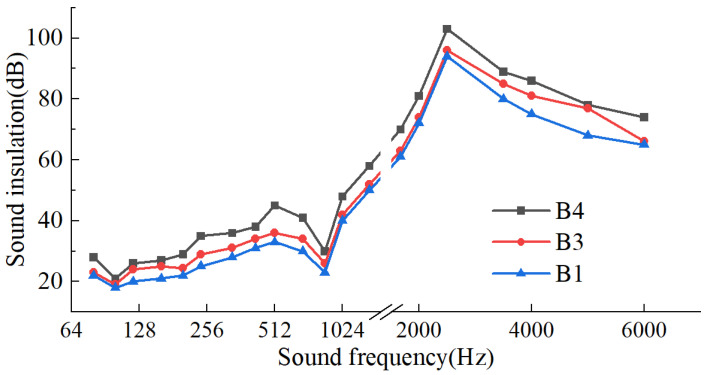
Isolation capability of wall panels with different thickness.

Figure 14 shows the sound insulation performance of wall panels with core layer thicknesses of 1 mm (B1), 2 mm (B2), and 3 mm (B3). It is evident that the thickness of these lightweight wall panels considerably influenced their sound insulation performance. The variations in sound insulation between the panels, at each sound wave frequency, spanned approximately 4 dB to 8 dB. When the sound wave frequency was in the low-to-mid-frequency range (64~1600 Hz), a thicker core layer effectively improved sound insulation at resonance frequencies. Additionally, increasing the core layer thickness not only reduced the critical frequency at which the coincidence effect occurs but also amplified the blocking capability of high-frequency sound waves.

(2) Effect of core layer sequence on sound insulation performance

**Figure 15:**
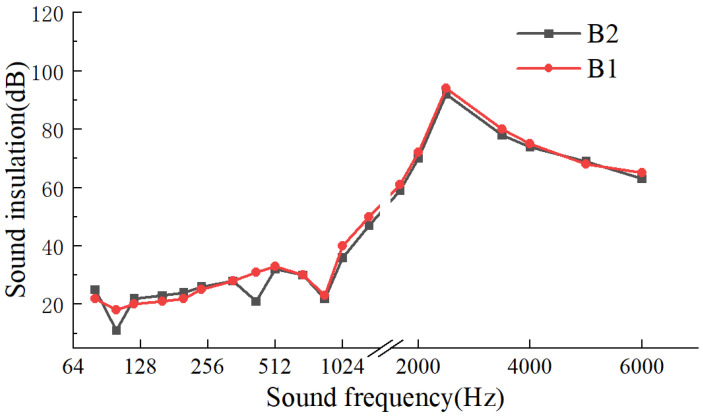
Effect of core layer sequence on sound insulation performance.

Figure 15 presents the test results of B1 and B2, demonstrating the effect of core layer sequence on the sound insulation performance of lightweight wall panels. In the high-frequency range of sound waves (>1600 Hz), changes in the sequence of core layers in the lightweight wall panels had minimal impact on sound insulation performance. Within the frequency range of 64~700 Hz, excluding the resonance points, six frequency bands were selectively chosen for testing the average sound insulation performance. It was found that the transmission loss of B1 and B2 panels did not exceed 3 dB. Therefore, the sound insulation performance of lightweight wall panels was not affected by the sequence of core layers.

(3) Influence of outer panels on sound insulation performance

**Figure 16:**
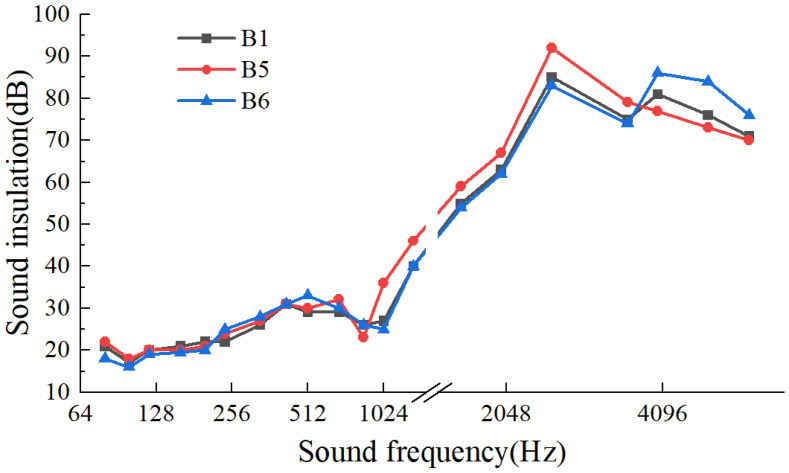
Influence of outer panels on sound insulation performance.

Figure 16 illustrates the impact of different panel materials on the sound insulation performance of recycled lightweight wall panels. In the low-to-mid-frequency range (20~200 Hz), the panel material exhibited minimal influence on sound insulation, with variations between 1 dB and 3 dB. The ranking of impact was B5 > B1 >B6. The sound insulation performance of aluminum and steel panels surpassed that of wood panels, attributed to the higher rigidity and stiffness of metal materials. In the mid-to-high-frequency range (200~3500 Hz), the sound insulation performance was divided into the resonance phase and the density phase. During the resonance phase, metal panels demonstrated a reduction in sound insulation performance due to the resonance effect, with the ranking being B6 > B1 > B5. In the density phase, stainless steel panels, featuring the highest density, exhibited a linear increase in sound insulation performance with frequency, altering the ranking to B5 > B1 > B6. When the frequency exceeded 4000 Hz, the overall sound insulation performance decreased, with the ranking being B6 > B1 > B5. Wood panels, owing to their higher modulus of elasticity and superior damping properties, exhibited better sound insulation performance in the high-frequency range in comparison to metal materials.

### 4.2. Numerical Simulation of Sound Insulation Performance of Wall Panels

#### 4.2.1. Numerical Simulation Experimental Program

**Figure 17:**
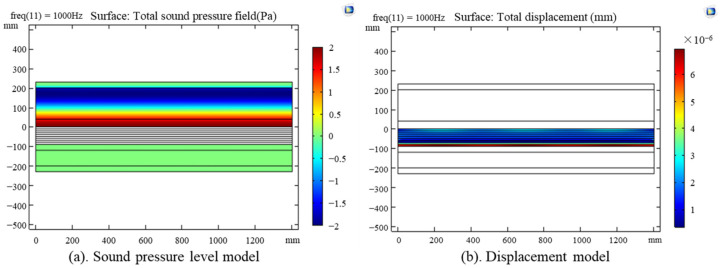
Two-dimensional simulation model of wall panels: (**a**) sound pressure model, (**b**) displacement model.

This study employed COMSOL 5.5 (COMSOL Inc., Stockholm, Sweden) Multiphysics modeling and simulation software for multi-physical field analysis [37]. A two-dimensional reverberation chamber, based on the finite element method, served as the simulation field for the lightweight wall panel model, with a one-third octave band sound source module chosen for the experiment. During modeling, the core layer’s density, flow resistance, elastic modulus, and Poisson’s ratio were set as parameters. The sound insulation performance of recycled lightweight wall panels was simulated by solving partial differential equations. The sound insulation performance of the wall panels was determined by calculating the sound pressure level difference and displacement amplitude difference between the walls, as illustrated in Figure 17. 

#### 4.2.2. Simulation Analysis of Sound Insulation Performance

(1) Effect of core thickness

**Figure 18:**
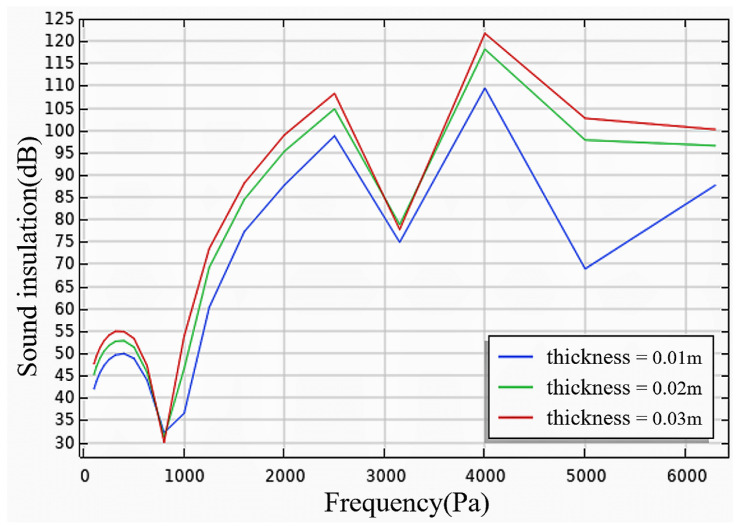
Simulation of sound insulation performance of wall panels with different thicknesses.

The experimental parameters were entered into the COMSOL software, with the core layer thickness set to 3 cm, 2 cm, and 1 cm. As depicted in Figure 18, the simulated sound insulation performance of the foam concrete core layer aligns with the experimental results. In the low-to-mid-frequency range (20~2000 Hz), the simulated sound insulation was slightly higher, likely due to differences between the test and the simulation environment. In the high-frequency range (>2000 Hz), the simulated sound insulation was slightly lower, and the matching effect was inconsistent. This inconsistency may stem from increased power due to sound wave superposition in the standing wave tube method test, or irregular material distribution in the actual experiment. When the sound frequency surpassed 3500 Hz, the simulated sound insulation initially increased and subsequently decreased. This was because as the sound wave frequency increases, the wavelength becomes smaller than the core layer thickness, causing most sound waves to pass directly through, thereby reducing sound insulation.

(2) Effect of core layer sequence

**Figure 19:**
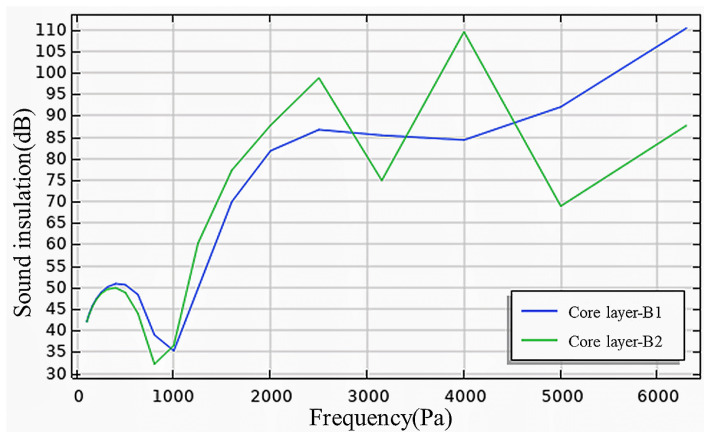
Simulation of sound insulation performance of different core layer sequences.

Figure 19 presents the numerical simulation results for B1 and B2. In the low–to-mid-frequency range, the experimental results were generally consistent with the simulation outcomes, with deviations stemming from environmental discrepancies. When the sound wave frequency surpassed 2500 Hz, notable differences emerged between the experimental and simulation results for different core layer sequences, attributed to the same factors previously discussed. Altering the sequence revealed that the coincidence effect of the wall panels was not significant, and sound insulation progressively increased with frequency once the sound frequency exceeded 4000 Hz. The error primarily arose from the simulation setting the porosity of each core layer to be identical and placing denser core layers in the front, which reduces overall sound insulation performance. In actual production, variations in the porosities of the core layers contributed to these disparities. Overall, the core layer sequence had a minimal impact on the sound insulation performance of recycled lightweight wall panels.

(3) Impact of different material panels on sound insulation performance

**Figure 20:**
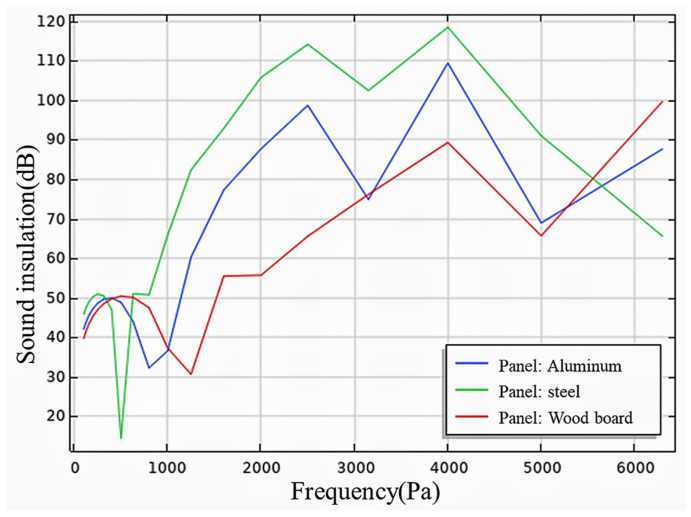
Impact of different material panels on sound insulation performance.

Figure 20 displays the numerical simulation results for B1, B5, and B6. The simulation results were generally consistent with the actual performance, with numerical discrepancies primarily occurring in the low- and mid-frequency ranges. These discrepancies can be attributed to differences between the experimental and simulation environments.

## 5. Conclusions

This study explored the utilization of municipal solid waste incineration bottom ash (MSWIBA) in producing foam concrete by activating MSWIBA from municipal solid waste incineration facilities and incorporating it as a filler in lightweight wall panels to achieve high-value-added utilization. The research process and primary conclusions were as follows:

(1) Using grey theory and mortar tests for optimization, the optimal fineness and dosage of MSWIBA were established. After adjusting the fineness of the micro-powder and its water demand, the maximum activity index increased from 64.1% to 90.1%. During the preparation of the recycled micro-powder, it was recommended to maintain a specific surface area of 800~900 m^2^/kg, with particle sizes below 3 μm and a maximum diameter not exceeding 75 μm, replacing 10~20% of the cement.
(2) Single-factor and orthogonal experiments were used to identify the influencing factors and determine the optimal mix ratio. The factors affecting the 28-day compressive strength, listed in order of impact, were apparent dry density, foaming agent dilution ratio, and water–cement ratio. For the water absorption rate, the order of influence was the foaming agent dilution ratio, apparent dry density, and water–cement ratio. The optimal parameters included an apparent dry density of 550 kg/m^3^, a water–cement ratio of 0.4, and a foaming agent dilution ratio of 1:20.
(3) As the thickness of the MSWIBA foam concrete core layer increased, the sound absorption and insulation performance of the wall panels improved, especially in the high-frequency range. The arrangement of the core layers had a minimal effect on acoustic performance. Wood panels displayed effective sound insulation within the 200~4000 Hz frequency range. The COMSOL simulation results closely matched the experimental data.

However, this study primarily considered external factors in the preparation of MSWIBA foam concrete and offers limited in-depth analysis. The influence of the internal structure of lightweight wall panels on acoustic performance and surface effects at the microscale will be further studied in the future.

## Figures and Tables

**Table 1:** MSWIBA surface area classification m^2^/kg.

C	P1	P2	P3	P4	P5	P6	P7	P8
361	300–400	400–500	500–600	600–700	700–800	800–900	900–1000	1000–1100

**Table 2:** The main chemical components of MSWIBA.

Element	SiO_2_	CaO	Al_2_O_3_	Na_2_O	Fe_2_O_3_	P_2_O_5_	K_2_O	MgO	TiO_2_	Other
Content (%)	48.42	14.77	11.98	3.26	5.41	1.87	1.41	1.77	0.77	10.34

**Table 3:** Performance index of foaming agent.

Index	Foaming Multiple	Settlement Distance (mm)	Water Secretion (mL)
Parameter	>20	<10	<80

**Table 4:** Test scheme for strength of MSWIBA mortar with different specific surface areas.

Test Groups	Specific Surface Area	MSWIBA (g)	Cement (g)	Standard Sand (g)	Water (g)
N_a_-P1	P1	135	315	1350	225
N_a_-P2	P2	135	315	1350	225
N_a_-P3	P3	135	315	1350	225
N_a_-P4	P4	135	315	1350	225
N_a_-P5	P5	135	315	1350	225
N_a_-P6	P6	135	315	1350	225
N_a_-P7	P7	135	315	1350	225
N_a_-P8	P8	135	315	1350	225
N_a_-C1	-	0	315	1350	225
N_a_-C2	-	0	450	1350	225

**Table 5:** Test plan for different dosages of MSWIBA.

Test Groups	Replacement Rate	Cement (g)	Standard Sand (g)	Water (g)
D1	5%	427.5	1350	225
D2	10%	405	1350	225
D3	15%	382.5	1350	225
D4	20%	360	1350	218.4
D5	25%	337.5	1350	210
D6	30%	315	1350	201.6
D7	35%	292.5	1350	193.2
D8	40%	270	1350	184.8
D9	45%	247.5	1350	176.4
D10	50%	225	1350	168
D11	0%	450	1350	225

**Table 6:** Apparent dry density mix proportion.

Num	Dry Density (kg/m^3^)	Cement (g)	MSWIBA (g)	Foaming Agent (g)	Dilution Multiple	Water–Cement Ratio
α1	300	1125	125	4.55	1:40	0.45
α2	350	1312.2	145.8	4.49	1:40	0.45
α3	400	1500.3	166.7	4.42	1:40	0.45
α4	450	1687.5	187.5	4.35	1:40	0.45
α5	500	1875.0	208.3	4.28	1:40	0.45
α6	550	2062.5	229.2	4.21	1:40	0.45
α7	600	2250	250	4.15	1:40	0.45

**Table 7:** Mix proportion of water–cement ratio.

Num	Dry Density (kg/m^3^)	Cement (g)	MSWIBA (g)	Foaming Agent (g)	Dilution Multiple	Water–Cement Ratio
β1	500	1875.0	208.3	4.50	1:40	0.4
β2	500	1875.0	208.3	4.29	1:40	0.45
β3	500	1875.0	208.3	3.80	1:40	0.5
β4	500	1875.0	208.3	3.56	1:40	0.55
β5	500	1875.0	208.3	3.44	1:40	0.6

**Table 8:** Foaming agent dilution ratio.

Num	Dry Density (kg/m^3^)	Cement (g)	MSWIBA (g)	Foaming Agent (g)	Dilution Multiple	Water–Cement Ratio
γ1	500	1875.0	208.3	8.39	1:20	0.45
γ2	500	1875.0	208.3	4.29	1:40	0.45
γ3	500	1875.0	208.3	2.89	1:60	0.45
γ4	500	1875.0	208.3	2.18	1:80	0.45

**Table 9:** Mix proportion of admixtures.

Num	Dry Density (kg/m^3^)	Cement (g)	MSWIBA (g)	Foaming Agent (g)	Dilution Multiple	Water–Cement Ratio	Admixtures (g)
Test group	500	1875.0	208.3	4.29	1:40	0.45	2.5% Na_2_CO_3_ + 0.5% water reducer + 0.05% triethanolamine
Control group	500	1875.0	208.3	4.29	1:40	0.45	-

**Table 10:** Multi-factor orthogonal mix proportion.

Num	Apparent Dry Density (α)	Water–Cement Ratio (β)	Foaming Machine Dilution Multiple (γ)
1	350 kg/m^3^	0.4	1:20
2		0.45	1:40
3		0.5	1:60
4	450 kg/m^3^	0.4	1:40
5		0.45	1:60
6		0.5	1:20
7	550 kg/m^3^	0.4	1:60
8		0.45	1:20
9		0.5	1:40

**Table 11:** Grey correlation between mortar strength and particle size distribution.

	X1 (%) (0.5~5 μm)	X2 (%) (5~20 μm)	X3 (%) (20~75 μm)	X4 (%) (75~200 μm)
Y1	+0.765	+0.8701	−0.664	−0.4458
Y2	+0.7606	+0.8514	−0.7244	−0.4613

**Table 12:** Experiment on fluidity of mortar.

Num	Cement (g)	MSWIBA (g)	Sand (g)	Water Consumption (mL)	Diffusion Diameter (mm)	Vertical Diameter (mm)	Average Value (mm)
1	450	0	1350	225	224.1	223.6	223.8
2	315	135	1350	225	239.1	239.3	239.2
3	315	135	1350	179.1	-	-	-
4	315	135	1350	199.1	179.1	179.1	179.1
5	315	135	1350	204.1	204.1	204.2	204.1

**Table 13:** Mortar strength activity index with different MSWIBA specific surface area.

Test Group	Water Consumption	P1	P2	P3	P4	P5	P6	P7	P8	C1	C2
N_a_	225 g	43.4	50.3	53.4	54.0	64.9	53.8	51.4	51.9	61.4	100
N_b_	201.6 g	45.5	57.0	72.6	71.8	66.6	62.1	74.9	53.8	47.8	100

**Table 14:** Mortar strength activity index with different dosage of MSWIBA.

Test Group	D1 (5%)	D2 (10%)	D3 (15%)	D4 (20%)	D5 (25%)	D6 (30%)	D7 (35%)	D8 (40%)	D9 (45%)	D10 (50%)	D11 (0%)
Activity Index (%)	90.1	88.8	76.9	72.2	73.3	65.7	63	31.8	23.6	22.9	100

**Table 15:** Effect of admixtures.

Test Group	28d-Compressive Strength/MPa
Without additives	2.3
Add admixture	2.9

**Table 16:** Orthogonal test result.

Num	1	2	3	4	5	6	7	8	9
28d-compressive strength (MPa)	2.6	1.6	1.5	2.1	1.8	2.5	2.3	3.1	2.9
Water absorption (%)	10.2	12.1	19.9	13.8	17.1	7.2	15.5	7.8	11.6

**Table 17:** Experimental analysis of range.

Factor	Sum of Indicators	α	β	γ
		K_α_	K_β_	K_γ_
28d-compressive strength (MPa)	K_1_	5.7	7.0	7.0
	K_2_	6.4	6.5	5.6
	K_3_	7.3	5.9	5.6
	k_1_	1.90	2.33	2.33
	k_2_	2.13	2.17	1.87
	k_3_	2.43	1.97	1.87
	R	1.6	1.1	1.4
	Importance	αγβ		
	Best plan	α_3_γ_1_β_2_		
Water absorption (%)	K_1_	42.2	39.5	29.0
	K_2_	38.1	37.0	37.5
	K_3_	34.9	38.7	52.5
	k_1_	14.07	13.17	9.67
	k_2_	12.70	12.33	12.50
	k_3_	11.63	12.90	17.50
	R	7.3	2.5	15
	Importance	γαβ		
	Best plan	γ_1_α_3_β_2_		

**Table 18:** Optimum mix proportion.

Parameter	Dry Density (kg/m^3^)	Cement (g)	MSWIBA (g)	Foaming Agent (g)	Dilution Multiple	Water–Cement Ratio	Admixtures (g)
Index	550	2062.5	229.2	8.24	1:20	0.4	2.5% Na_2_CO_3_ + 0.5% water reducer + 0.05% triethanolamine

**Table 19:** Test plan of wallboard.

Num	Number of Layers	Panel Layer Material	Core Layer Sequence	Core Thickness (mm)
B1	5	Aluminum	(v)(iv)(iii)(ii)(i)	1
B2		Aluminum	(v)(iii)(ii)(iv)(i)	1
B3		Aluminum	(v)(iv)(iii)(ii)(i)	2
B4		Aluminum	(v)(iv)(iii)(ii)(i)	3
B5		Stainless steel	(v)(iv)(iii)(ii)(i)	1
B6		Board	(v)(iv)(iii)(ii)(i)	1

## Data Availability

All relevant data are within the paper.
